# Circulating circRNA predicting the occurrence of hepatocellular carcinoma in patients with HBV infection

**DOI:** 10.1111/jcmm.15635

**Published:** 2020-07-21

**Authors:** Chen Wu, Lei Deng, Han Zhuo, Xiang Chen, Zhongming Tan, Sheng Han, Junwei Tang, Xiaofeng Qian, Aihua Yao

**Affiliations:** ^1^ Hepatobiliary Center Key Laboratory of Liver Transplantation The First Affiliated Hospital of Nanjing Medical University Chinese Academy of Medical Sciences NHC Key Laboratory of Living Donor Liver Transplantation (Nanjing Medical University) Nanjing China

**Keywords:** AFP, biomarker, chronic hepatitis, circRNA, extracellular

## Abstract

A microarray‐based high‐throughput screening of human circulating circular RNA (circRNA) was applied with five patients newly diagnosed with hepatocellular carcinoma (HCC), five patients with HBV‐positive chronic hepatitis (CH) and five healthy controls (NC) enrolled. The plasma of HCC patients after hepatectomy was also collected. After multiple staged validation, we obtained five circRNAs as candidate. Based on the stratified risk score analysis, three increased circRNAs including circ_0009582, circ_0037120 and circ_0140117 were confirmed as candidate circulating fingerprints for distinguishing HCC from CH or NC group. With the combination of AFP, higher sensitivity and specificity were further guaranteed, suggesting that circ_0009582, circ_0037120 and circ_0140117 may serve as potential biomarkers for predicting the occurrence of HCC in patients with HBV infection.

## 1 INTRODUCTION

As a new member of human endogenous non‐coding RNA, the circular RNA, also defined as circRNA, is becoming a research hotspot in the field of RNA, attracting a lot of attention.^1^, ^2^ The potential function of circRNA has been identified in multiple human molecular biological events such as cell proliferation, differentiation and invasion.^3^, ^4^ Meanwhile, the cell singling transduction, cell‐cell crosstalk and the cell metabolism were also participated.^5^, ^6^ The major mechanism for circRNA was revealed by acting as the sponge, interacting with certain mRNA and miRNA, which was further identified as competing endogenous RNA (ceRNA).^3^, ^7^

Recently, the existence of circRNA in human body fluid was identified, this bring a new filed for the potential function for circRNAs as fingerprints in human disease, especially in human malignant cancers.^8^, ^9^ For example, researchers have proved circ‐KIAA1244 could serve a novel circulating biomarker for detection of gastric cancer.^1^ The circRNAs, circRNA_0001178 and circRNA_0000826, may serve as a potential diagnostic biomarker for liver metastases from colorectal cancer.^2^ However, to our knowledge, the biomarker‐based investigation focusing on circRNA in human hepatocellular carcinoma (HCC) with the HBV infection background in Chinese Han population was poor investigated.

In this study, we revealed the landscape of human circulating circRNA through the microarray technology in HCC patients before and after operation, patients with HBV‐associated CH, and healthy controls. The diagnostic value of circRNA as biomarker was estimated by the multiple staged validation and stratified risk score analysis.

## 2 MATERIALS AND METHODS

### 2.1 CircRNA microarray

The CircRNA array was designed by CapitalBio Technology by using the Human CircRNA Array version platform. This array contains probes targeting about 170 340 human circRNAs. Those circRNA target sequences were all from Circbase, Deepbase and Rybak‐Wolf 2015. All the results were confirmed by multiple quality control by the manufacturer. Microarray data are available in the ArrayExpress database (www.ebi.ac.uk/arrayexpress) under accession number E‐MTAB‐8799.

### 2.2 Patients and samples

Patients enrolled in this study were diagnosed with HCC in The First Affiliated Hospital of Nanjing Medical University between September 2012 and May 2017. All the blood samples were collected before the surgical operation, separated immediately and collected in a separate vacuum cube, followed by centrifugation at 800 *g* for 10 minutes. All the samples collected were according to the Helsinki Declaration of 1975, as revised in 2008. The informed consent was obtained from all the participants of this study. This study was approved by the Institutional Review Board of Nanjing Medical University. For all the participants enrolled in each group, age and sex were well matched (Table S1).

### 2.3 RNA isolation

TIANamp Virus RNA Kit with TRIzol reagent (Thermo Fisher Scientific) was used for total RNAs isolation. The synthetic *C elegans* miRNA (cel‐miR‐39, Applied Biosystems) was added to each sample as external reference. The cDNA was generated by using the Super‐Script First‐Strand Synthesis System (Invitrogen). Five microgram of DNase‐treated total RNA was incubated with or without 7.5 unit of RNase R (Lucigen, Catalog No. RNR07250) for 10 minutes at 37°C. RNA was purified and concentrated as described previously before subjecting to RT‐PCR or RT‐qPCR analysis. The experiment was repeated two additional times, and three biological replicates of the samples were analysed. Meanwhile, the GAPDH was used for the internal normalization. The detailed primer was presented in Table S4.

### 2.4 Risk score analysis

All the samples were applied with training set and validation set. For training set, 20 samples were enrolled with randomly selection. The algorithms comparative 2^‐ΔΔCt^ method was applied for analysing.

Risk score analysis was a traditional analysis for validating a certain biomarker. Here data in the training set was performed to investigate the associations between the concentrations of the plasma circRNA expression levels. The upper 95% reference interval of each circRNA value in controls was set as the threshold to code the expression level of the corresponding circRNA for each sample as 0 and 1 in the training set. A risk score function (RSF) was defined according to a linear combination of the expression level for each circRNA. For example, the RSF for sample i using information from circRNAs was rsfi = ∑3j‐1Wj.sij. In the above equation, sij is the risk score for circRNA j on sample i, and Wj is the weight of the risk score of circRNA j. We conducted the ROC analysis by using the total RSF value according to the case‐control group in training set. We chose the value as the cut‐off value because the value of sensitivity + specificity was maximal.

### 2.5 Statistical analysis

If no special, data were presented as mean ± SD. The classification variables were counted by chi‐square test. Continuous variables were counted by Student's *t* test. Statistical analysis was performed using STATA 10.0 and presented with GraphPad Prism 5.0. Results were considered statistically significant at *P* < .05.

## 3 RESULTS

### 3.1 Identification of differentially expressed circRNAs

**FIGURE 1:**
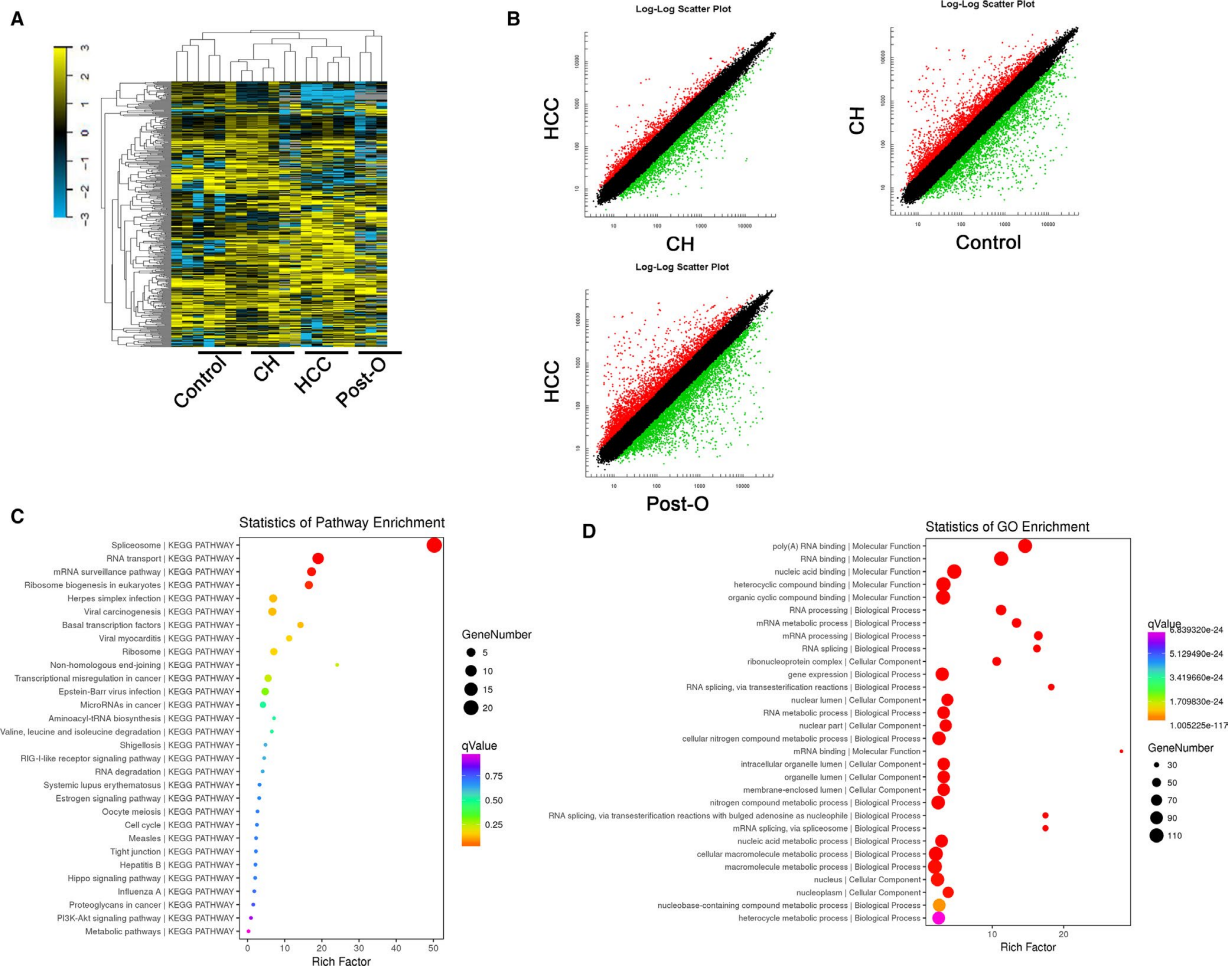
The expression landscape of circulating circRNAs in HCC patients and matched control groups. A, Cluster analysis of the different expression of the circRNAs extracted from plasma in different groups. B, Volcano plot shows the up‐regulated and down‐regulated circRNAs in different groups. Higher expression levels are indicated by ‘red’, lower expression levels are indicated by ‘green’, and no significant difference is indicated by ‘black’. C, KEGG analysis of circRNAs in different groups. D, GO analysis of circRNAs in different groups. HCC, hepatocellular carcinoma; CH, chronic hepatitis; Control: normal control; Post‐O, the paired HCC patients after operation

The circRNA microarray was applied to detect the circRNA expression landscape in plasma of five new diagnosed HCC patients (HCC group), and matched plasma of post‐operation was also detected (HCC post‐O group). Another five patients with HBV‐positive chronic hepatitis (CH) and five normal individuals were detected as control group. Cluster analysis presenting with heatmap presented a different expression profile among these four groups (Figure 1A). Paired comparing indicated the statistically significant circRNA in HCC patients pre‐ and post‐operation, HCC patients comparing with CH patients, CH patients comparing with healthy controls by scatter plots between the two groups were identified with the following parameters: fold changes ≥ 2.0 and *P* value ≤ .05, results were shown in Figure 1B, indicating a remarkable different expression landscape of circRNAs in four groups. Bioinformatic analysis by using GO, KEGG and disease pathway analysis suggested that these differentially expressed circRNAs were relevant to several vital physiological processes, cellular components, molecular functions and critical signalling pathways (Figure 1C,D).

### 3.2 Validation of differentially expressed circRNAs from training set and validation set

**FIGURE 2:**
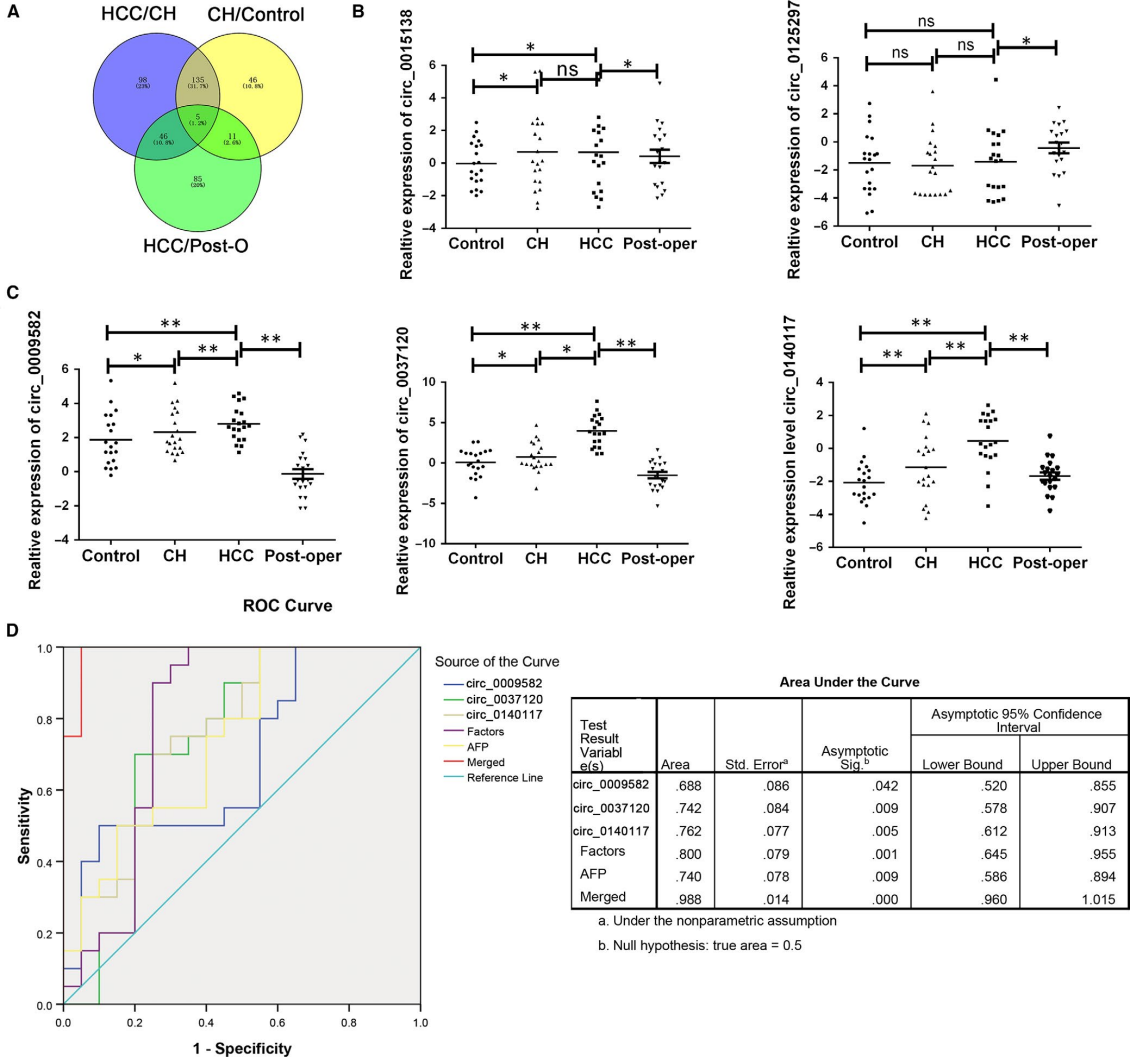
The screening work for candidate circulating circRNA in training set. A, Venny analysis of differently expressed circRNA in HCC, CH, NC and Post‐O groups. B, The expression of circ_0015138 or circ_0125297 was confirmed by RT‐PCR in groups. C, The expression of circ_0009582, circ_0037120 and circ_0140117 was confirmed by RT‐PCR in groups. Data were presented as plot of the mean with SD with log‐transformed. D: The ROC curve for three‐potential fingerprint in training set. *indicated *P* < .05, **indicated *P* < .01, n.s. indicated no significance. HCC, hepatocellular carcinoma; CH, chronic hepatitis; Control, normal control; Post‐oper, the paired HCC patients after operation

Based on the initial objective for biomarker screening, we tend to focus on the increased circRNAs in HCC groups. From the total 55 384 circRNAs detected, 284 circRNAs were significant up‐regulated in HCC patients compared with CH group. A total of 197 circRNAs were increased in CH patients compared with the healthy participants. Among the 140 rapid increased circRNAs, only five circRNAs presented a decreased level after operation, which was consistent with the secretion hypothesis by certain biomarker. The following five circRNAs including circ_0015138, circ_0125297, circ_0009582, circ_0037120 and circ_0140117 were labelled as candidates (Figure 2A). All the parameters were first detected in the training data set and then confirmed in the validation set. The 20 samples in training set were randomly selected. We further examined these differentially expressed circRNAs by RT‐qPCR in all the samples we enrolled. As presented in Figure 2B, neither circ_0015138 nor circ_0125297 presented a perfect significant in grouped comparing while circ_0009582, circ_0037120 and circ_0140117 presented an increased level in HCC comparing with either CH or control group and decreased after the hepatectomy (Figure 2C). After calculated, we obtained the three circRNA expression in the training set. Next, we compared the ability of the three circRNAs in predicting HCC from healthy controls or CH patients. ROC curve analysis was applied to investigate the diagnostic sensitivity and specificity for HCC. The separated circRNA and three merged factors were analysed, respectively. AFP as the traditional biomarker for HCC with low specificity was also enrolled. As presented in Figure 2D, the areas under the curve (AUC) of circ_0009582, circ_0037120 and circ_0140117, combination of circRNAs (Factors), AFP, and combination of circRNAs and AFP (Merged) were 0.688, 0.742. 0.762, 0.800, 0.740 and 0.988, respectively, in training set.

**FIGURE 3:**
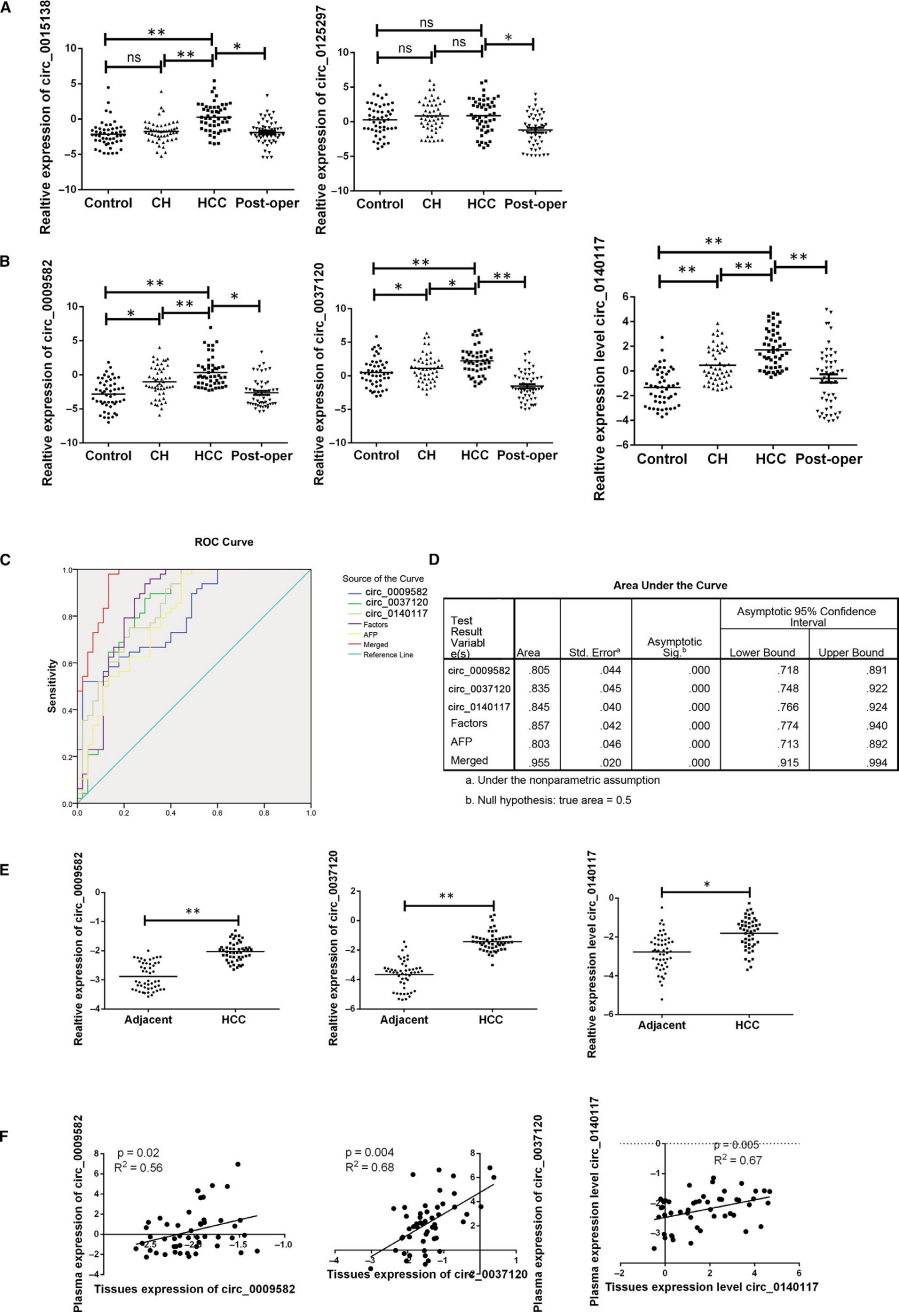
Predicting ability for candidate circRNA in validation set. A, The expression of circ_0015138 or circ_0125297 was confirmed by RT‐PCR in groups. B, The expression of circ_0009582, circ_0037120 and circ_0140117 was confirmed by RT‐PCR in groups. Data were presented as plot of the mean with SD with log‐transformed. C and D, The ROC curve for three‐potential fingerprint in training set. E, Relative expression of three circRNA in tissues samples. F, Pearson correlation in plasma sample and tissues samples. *indicated *P* < .05, **indicated *P* < .01, and n.s. indicated no significance. HCC, hepatocellular carcinoma; CH, chronic hepatitis; Control: normal control; Post‐oper: the paired HCC patients after operation

For the validation set, the similar analysis was employed. As presented in Figure 3A, circ_0015138 or circ_0125297 was confirmed with no significance while the increased level of circ_0009582, circ_0037120 and circ_0140117 was consistent with training set (Figure 3B). We also performed the risk score analysis in validation set with ROC curve. The of circ_0009582, circ_0037120 and circ_0140117, combination of circRNAs (Factors), AFP, and combination of circRNAs and AFP (Merged) were 0.805, 0.835. 0.845, 0.857, 0.803 and 0.955 respectively in validation set (Figure 3C,D).

According to the cut‐off which was defined as the maximal value of sensitivity plus specificity, we calculated the positive predictive value (PPV) and negative predictive value (NPV). For distinguishing HCC from controls, PPV and NPV were 95% and 95% in the training set, respectively. The same value was applied in validation sets, and the PPV and NPV were presented as 80% and 95%, respectively (Table S2). For predicting HCC from CH patients, the PPV and NPV in training set were 90% and 95% while in validation set were 84% and 80%, respectively (Table S3).

To better answer why these circRNAs were overpresented in HCC patient blood samples, we further examined the candidate circRNA expression levels in HCC tissue and their corresponding non‐tumorous part to find the potential correlations between the tumour tissue and peripheral blood samples. The increased level of the three circRNAs was revealed in tumour tissues of HCC patients compared with the adjacent tumour tissues. Meanwhile, we also investigated the correlation of the three circRNA expression in plasma samples and tissues sample, and a positive correlation was also obtained indicating that the increased circRNA might origin from the tumour (Figure 3E,F).

### 3.3 Stability expression of circRNAs

The expression of the three circRNAs was detected in RNA sample extracted from four healthy controls and was incubated at room temperature for 12 hours and 24 hours, subjecting it to up to five cycles of freezing and thawing or under storage of −80°C for about 7 days. All the process had minimal effects on the concentrations of the circRNAs, demonstrating that these circRNAs might stable expressed in human body fluid (Figure S1).

## 4 DISCUSSION

Hepatocellular carcinoma is one of the most common malignancies worldwide, and it was identified with high mortality. In the past decades, researchers have explored the potential biomarker for HCC and various biomarkers either extracted from tumour tissues or cell‐free plasma have been reported as predicting the tumorigenesis, metastasis or prognosis of HCC patients.^10^, ^11^ For example, Tang et al investigated 300 HCC patients, and they found that among the pathologically confirmed HCC, the positive rate of AFP was relatively low.^12^ Besides, the circulating non‐coding RNA including miRNA and lncRNA have proved might be applied for biomarkers for multiple human cancers including bladder cancer, colorectal cancer and bladder cancer.^1^, ^13^, ^14^, ^15^ All the above evidence indicated that the exploration focusing on the non‐coding RNA as potential diagnostic measures for HCC is necessary.

Circulating ncRNAs have recently emerged as novel biomarkers of cancer development and progression. The first star for ncRNAs as biomarker was miRNA, and it has been identified in multiple human cancer and has been applied in clinical diagnosis.^16^, ^17^ Based on the important function of miRNA, the long non‐coding RNA, also known as lncRNA, was also a novel biomarker in human disease; for example, HOTAIR and MALTA have been used in a further clinical exploration.^18^, ^19^, ^20^ Recently, researchers are focusing on the prediction ability of circRNA in human cancers. There were increasing evidences indicated that circRNAs were involved in the development and progression of diseases, especially cancer. Recently, more attention was focused on the clinical cancer diagnostic value of circRNAs. Xu et al identified the circRNA_0000826 may serve as a potential diagnostic biomarker for liver metastases from colorectal cancer based on a RNA sequencing study. In addition, Tang et al also found that GC‐tissue‐derived circ‐KIAA1244 could serve a novel circulating biomarker for detection of GC. The clinical significance for our study could be descripted into two bullets. The first was we found the aberrant expressed circRNA in plasma samples of HCC patients which indicated that these circRNA might be applied for further biomarker for predicting HCC in HBV‐positive patients especially for these patients suffering from CH. Second, the early detection or diagnosis for HCC means early treatment, early operation, this could increase the prognosis for HCC patients. For further application, a lot of work still need to be conducted, for example the endogenous expression of circRNAs and the copy number of circRNA in plasma samples. However, in future, we might inform the non‐HCC patients with HBV infection background, he/she presented high risk of HCC due to the high expression of the three circRNA in their plasma samples.

In this study, we employed a microarray‐based screening for the potential fingerprint for HCC from patients with HBV infection, especially from CH patients or liver cirrhosis. AFP, the traditional biomarker for HCC, has been proved with low specificity in predicted HCC patients from CH or patient with liver cirrhosis, and it has also been validated in this study. Thus, a biomarker which could distinguish HCC from healthy controls as well as the HBV‐associated CH or liver cirrhosis was very crucial for clinical diagnosis. In this study, we obtained a higher sensitivity and specificity of the combination of circ_0009582, circ_0037120, circ_0140117 and AFP, indicating the three circRNAs, circ_0009582, circ_0037120 and circ_0140117, might be utilized to predict HCC from patients with CH or healthy controls. More in‐depth studies are required to confirm the potential mechanism of these circRNAs in the development of HCC.

## CONFLICT OF INTEREST

The authors declare no conflict of interest.

## AUTHOR CONTRIBUTION

**Chen Wu:** Conceptualization (equal); Data curation (equal); Investigation (lead). **Lei Deng:** Data curation (equal); Formal analysis (equal); Validation (equal). **Han Zhuo:** Investigation (equal); Methodology (equal); Validation (equal). **Xiang Chen:** Formal analysis (equal); Investigation (equal); Resources (equal). **Zhongming Tan:** Funding acquisition (equal). **Sheng Han:** Conceptualization (equal); Data curation (equal). **Junwei Tang:** Funding acquisition (equal). **Xiaofeng Qian:** Project administration (equal); Writing‐original draft (equal); Writing‐review & editing (equal). **Aihua Yao:** Conceptualization (equal); Data curation (equal); Project administration (equal); Supervision (equal); Visualization (equal); Writing‐review & editing (equal).

## Supporting information

App S1

## Data Availability

Microarray data are available in the ArrayExpress database (www.ebi.ac.uk/arrayexpress) under accession number E‐MTAB‐8799.
