# Supplementary material for: Circulating circRNA predicting the occurrence of hepatocellular carcinoma in patients with HBV infection
Source: J Cell Mol Med. 2020 Jul 21;24(17):10216–22. doi: 10.1111/jcmm.15635 (PMC7520265; doi:10.1111/jcmm.15635)
Supplement: Supplementary file 1 — App S1 [file JCMM-24-10216-s001.pdf]

Supplementary Files

Table S1. Clinicopathological features of surgical hepatocellular carcinoma (HCC), chronic hepatitis (CH) and cancer-free control samples.

Table S2. Risk score analysis of three circRNAs as fingerprints in HCC and cancer-free control plasma samples.

Table S3. Risk score analysis of three circRNAs as fingerprints in HCC and CH group.

Table S4. Primers for Quantitative RT- PCR

Supplementary Information 1. Detailed expression of circRNA

Supplementary Information 2. Risk score in all samples

Fig. S1. Expression stability examination of three circulating circRNAs. The circRNA obtained from three healthy controls plasma samples were incubated at room temperature for 12h, 24h or subjecting it to up to 3 cycles of freezing and thawing. Data were presented as plot of the mean with SD. No significant difference was observed in each group.

Table S1. Clinicopathological features of surgical hepatocellular carcinoma (HCC), chronic hepatitis (CH) and cancer-free control samples

	HCC	CH	Control	*P* value
**N**	200	200	200	
**Age Mean (SE) year**	55.43(12.4)	56.89(11.8)	56.79(11.6)	**0.33**[a]
**Sex (male/female)**	160/40	157/43	162/38	**0.28**[b]
**Differentiation grade**				
Well	92			
Moderate	55			
Poorly	53			
**Tumor Size(cm)**				
≤5cm	109			
>5cm	891			
**Tumor Number**				
Solitary	78			
Multiple	122			
**Tumor Capsular**				
Incomplete	12			
Complete	188			
**AFP(ng/mL)**	31.3(12.6)	29.2(10.3)	20.2(9.2)	**0.032**[a]
**TNM stage(I:II:III)**	94: 54: 52			
HBV				
Yes	200	200	/	**1.00**[b]
No	0	0	/	

[a] Student t-test.

[b] Chi-square test.

Table S2: Risk score analysis of in hepatocellular carcinoma HCC and cancer-free control plasma samples

Score	0-9.734	9.734-12.112	PPV [a]	NPV[b]
**Training set**			0.95	0.95
HCC	1	19		
Control	19	1		
**Validation set**			0.80	0.95
HCC	36	144		
Control	171	9		

[a] PPV, positive predictive value

[b] NPV, negative predictive value

Table S3: Risk score analysis of in hepatocellular carcinoma HCC and CH plasma samples

Score	0-6.778	6.778-8.332	PPV [a]	NPV[b]
**Training set**			0.90	0.95
HCC	2	18		
CH	19	1		
**Validation set**			0.84	0.80
HCC	29	151		
CH	144	36		

[a] PPV, positive predictive value

[b] NPV, negative predictive value

Table S4. Primers for Quantitative RT- PCR

Gene name	All Patients	Sequence
circ_0015138	Forward Primer	TGCAGAAGTTACCACAGGCA
	Reverse Primer	CTTGGAAGCCGCAGAGGAGG
circ_0125297	Forward Primer	GGTTAATTGGCAGATCCCAA
	Reverse Primer	GCGGTTAAGAAGCGATTTAG
Circ_0037120	Forward Primer	TGGGTTCTGATGTTAGTCTT
	Reverse Primer	GGCCACGAATTCATGCTAGT
Circ_0009582	Forward Primer	GAGTGGTGTTATCCCGAGAG
	Reverse Primer	GGGAATTGGTTCATCATGCG
Circ_0140117	Forward Primer	GACGACATTTCACCTCG
	Reverse Primer	CCCATTTTGACAGCTTGGAT
GAPDH	Forward Primer	GGAGCGAGATCCCTCCAAAAT
	Reverse Primer	GGCTGTTGTCATACTTCTCATGG

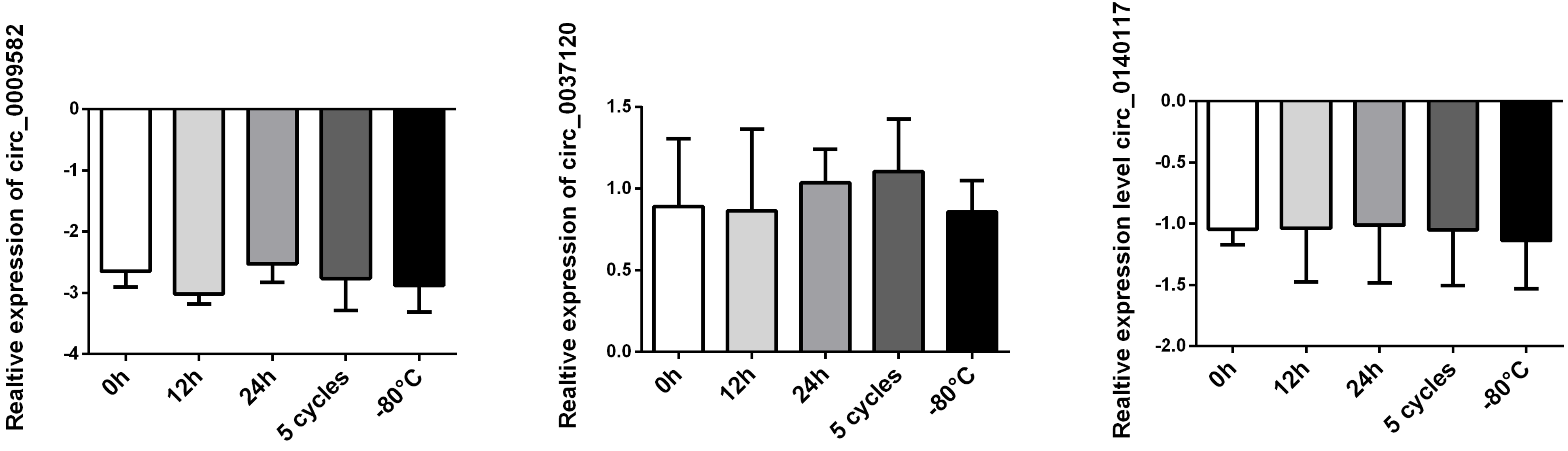

Realtive expression of circ_0009582
0
-1
-2
-3
-4
0h
12h
24h
5 cycles
-80°C
Realtive expression of circ_0037120
1.5
1.0
0.5
0.0
0h
12h
24h
5 cycles
-80°C
Realtive expression level circ_0140117
0.0
-0.5
-1.0
-1.5
-2.0
0h
12h
24h
5 cycles
-80°C
